# Case Report: Near-complete resolution of nail psoriasis with upadacitinib in a pediatric patient

**DOI:** 10.3389/fimmu.2026.1941127

**Published:** 2026-09-01

**Authors:** Yulin Huang, Sijian Wen

**Affiliations:** The First Affiliated Hospital of Guangxi Medical University, Nanning, China

**Keywords:** histopathological, nail psoriasis, onychomycosis, pediatric, upadacitinib

## Abstract

Nail psoriasis (NP) is refractory in children, causing punctate pits, onycholysis, subungual hyperkeratosis and discoloration, impairing quality of life. We report a 14-year-old boy with 4-year NP, all fingernails involved with no cutaneous lesion, and unresponsive to traditional therapy. After 20-week oral upadacitinib, all nail lesions achieved near-complete resolution with only minimal residual nail changes, and no treatment-related adverse events. This suggests that upadacitinib demonstrates favorable efficacy and safety for pediatric refractory NP, large-scale prospective clinical investigation are warranted to validate our findings.

## Introduction

In patients with psoriasis, approximately 80% have nail involvement, while nearly 6% present with nail involvement as the sole clinical manifestation (1). Approximately one-third of psoriasis cases have an onset in childhood, among which the prevalence of pediatric nail psoriasis ranges from 17% to 39.2% (2).

The clinical manifestations are primarily determined by the anatomic sites involved. Psoriasis affecting the nail matrix gives rise to nail pitting, leukonychia, red lunula, and nail dystrophy, whereas involvement of the nail bed results in subungual splinter hemorrhages, onycholysis, oil-drop sign (salmon patch), and subungual hyperkeratosis. Of these manifestations, the most common individual clinical features are onycholysis and nail pitting (3).

It has been confirmed that nail psoriasis induces physical and emotional distress, profound functional impairment, and diminished patient quality of life (QoL), necessitating timely management (4). The high keratin density within the nail plate impedes the percutaneous penetration of topical agents, frequently resulting in treatment failure (5). Thus, the management of nail psoriasis remains a major clinical challenge.

Pediatric psoriasis often necessitates sustained treatment extending into adulthood. To date, there are no consensus-based treatment guidelines for pediatric psoriasis. The safety profiles of certain medications in pediatric psoriasis patients remain unestablished, resulting in a more limited spectrum of therapeutic options compared with the adult population. In recent years, significant advances have been achieved in the management of cutaneous and nail psoriasis, owing to the advent of novel targeted therapies—particularly biologic agents, including etanercept, infliximab, adalimumab, ustekinumab, secukinumab, ixekizumab, and guselkumab—which represent a promising therapeutic modality for nail psoriasis (6). Herein, we present a clinical case of a 14-year-old male adolescent diagnosed with nail psoriasis who achieved full nail restoration after administration of upadacitinib, a selective Janus kinase 1 (JAK1) inhibitor.

## Case report

**Figure 1:**
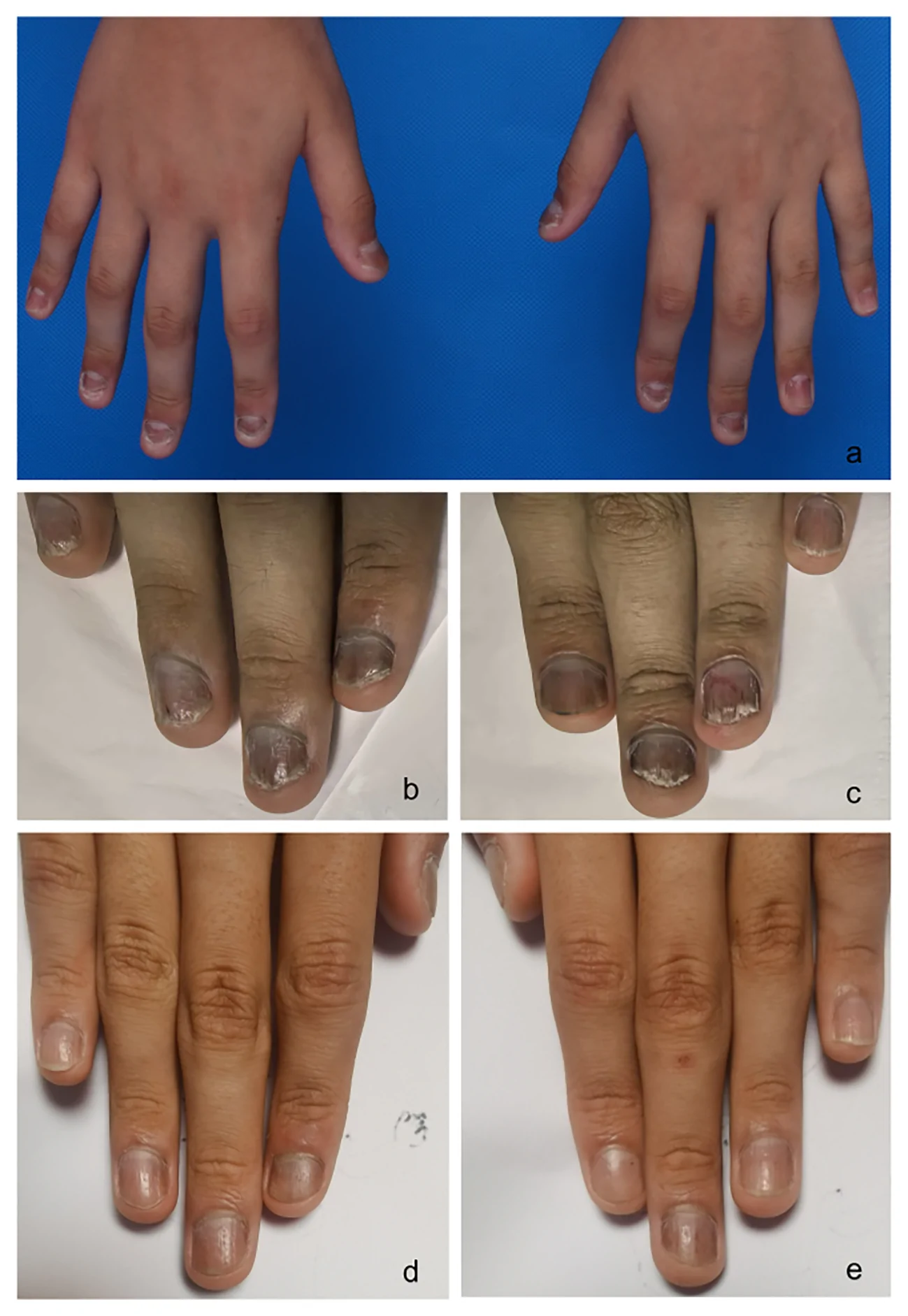
Pretreatment and posttreatment clinical image. **(a)** Initial clinical photographs of nail psoriasis at right hand. **(b, c)** The nail lesions showed significant improvement at 10 weeks. **(d, e)** At Week 20, nearly complete resolution of nail psoriasis was observed.

A 14-year-old male patient presented with a 4-year history of progressive nail lesions. All fingernails exhibited onychodystrophy, manifesting as varying degrees of nail plate thickening, crumbling, and melanonychia (**Figure 1a**). The toenails were unaffected. No systemic cutaneous pathological changes, such as erythema or squamous plaques, were observed upon physical examination. He denied any history of cutaneous lesions or arthralgia. The patient experienced severe discomfort, which compromised his academic performance and daily functional capacity. Based on these clinical features, onychomycosis or idiopathic nail dystrophy was initially suspected.

Though fungal microscopy and culture failed to detect any hyphae or spores, a preliminary diagnosis of onychomycosis was still made. The patient subsequently received a 3-month course antifungal regimen with itraconazole at a dosage of 0.2 g twice daily in August 2023; however, no significant clinical improvement was observed. Over the following years, the patient was prescribed additional systemic therapies, including cyclosporine, yet none of these interventions yielded meaningful clinical responses.

**Figure 2:**
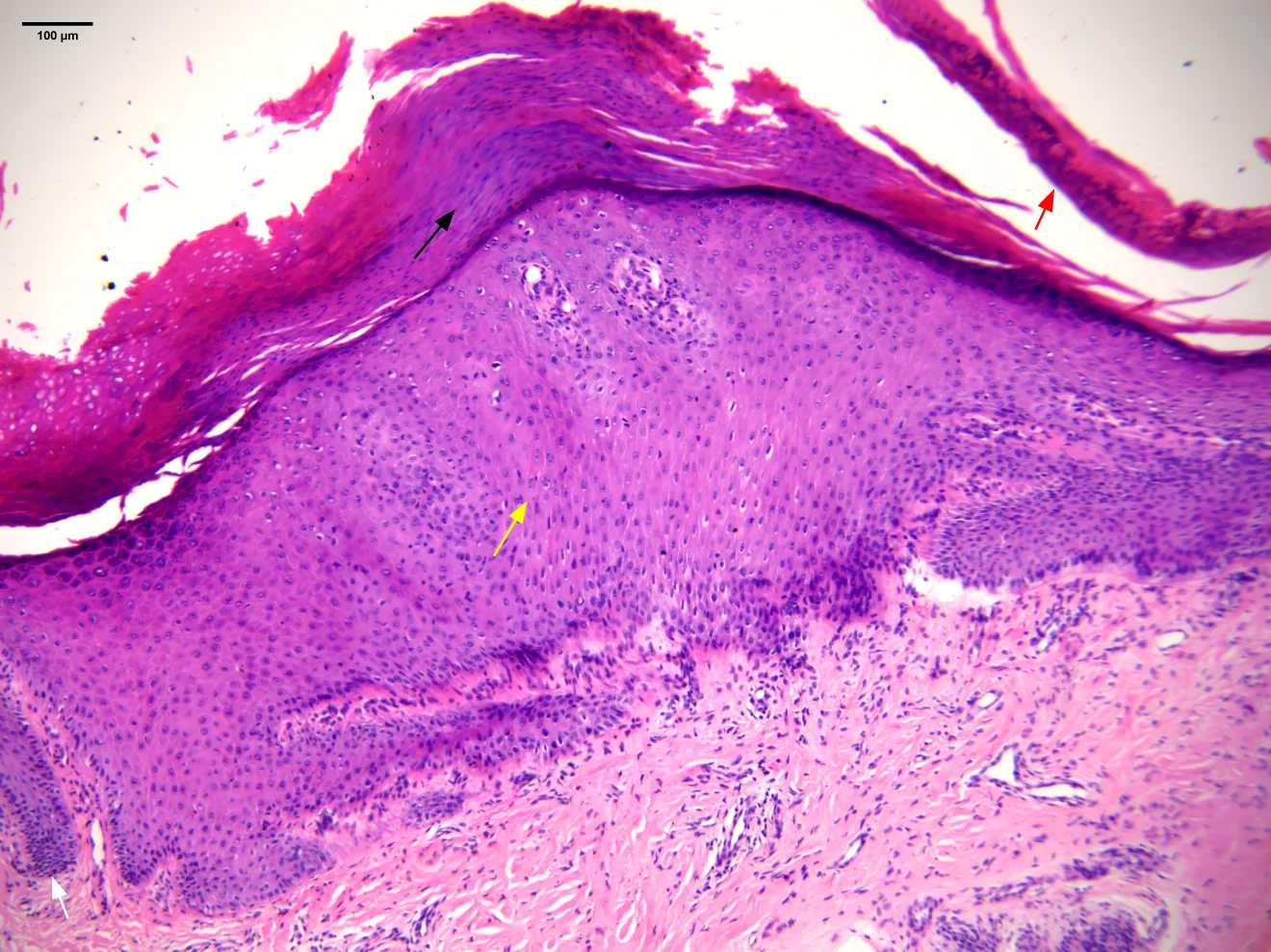
Nail biopsy. Nail plate hyperkeratosis accompanied by parakeratosis (black arrow), scattered neutrophil aggregates in the stratum corneum (red arrow), nail bed epithelial hyperplasia (yellow arrow) and hypertrophy with focal downward elongation of epithelial rete pegs (white arrow), partial epidermal spongiosis, as well as mild superficial dermal perivascular inflammatory cell infiltration. (HE staining: ×10).

Upon presentation at our outpatient clinic in July 2025, the patient underwent a nail biopsy. After routine cutaneous disinfection, full-thickness tissue was obtained via surgical excision from the proximal nail fold of the patient’s left thumb for standard histopathological evaluation. Histopathological examination of the nail specimen revealed hyperkeratosis with parakeratosis of the nail plate, a small number of neutrophil aggregates within the stratum corneum, hyperplasia and hypertrophy of the nail bed epithelium with focal downward extension of the epithelial pegs, foci of spongiosis, and mild perivascular inflammatory cell infiltration in the superficial dermis (**Figure 2**). Periodic acid-Schiff (PAS) staining yielded negative results. There was no family history of psoriasis, either at initial presentation or in retrospect after histopathological examination. Based on negative fungal laboratory findings and failure of standardized 3-month antifungal treatment, together with no history of nail trauma, alopecia, or pathognomonic skin-nail manifestations, other nail-damaging disorders including onychomycosis, traumatic nail dystrophy and alopecia areata-associated nail lesions were briefly excluded. Combined with characteristic nail manifestations and pathological results, a revised diagnosis of isolated nail psoriasis was established. A decision was made to start treatment with upadacitinib at a daily dosage of 15 mg in August 2025.

**Figure 3:**
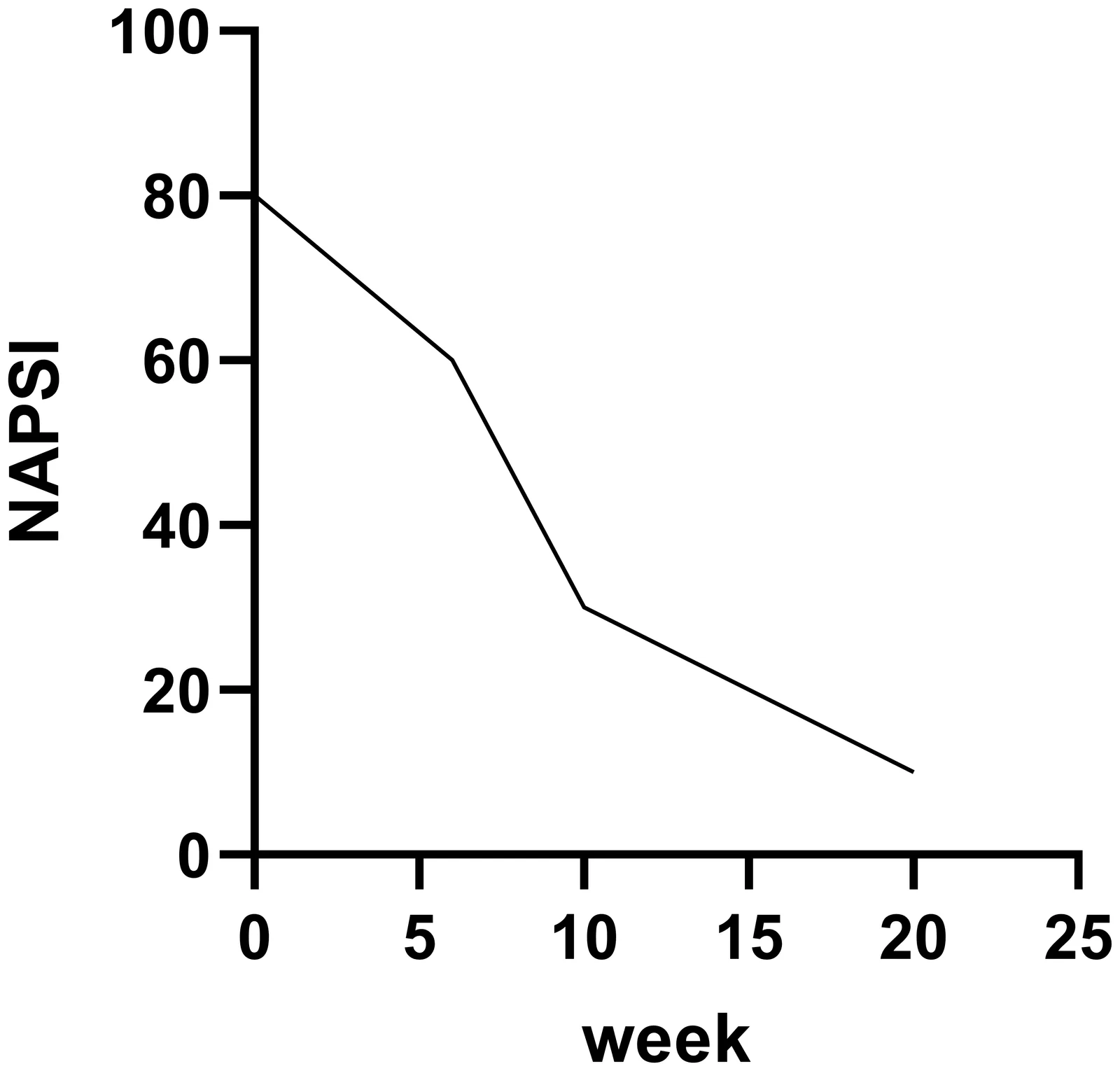
Serial NAPSI scores during the follow-up period. The NAPSI score was recorded as 80 at baseline, initial clinical improvement was noted at week 6, with the score decreasing to 60. By week 10, the score had declined further to 30, and near-complete resolution of nail lesions was achieved by week 20.

Initial clinical improvement was first observed at week 6 of continuous treatment. By week 10, reflecting a notable modification in nail psoriasis-related signs and symptoms, including nail pitting, onycholysis, and discoloration (**Figures 1b, c**). By week 20 of treatment, nearly complete resolution of psoriatic nail lesions was achieved across all affected fingernails; only a small number of nails exhibited mild residual pitting and faint discoloration (**Figures 1d, e**). During the treatment and follow-up period, serial dynamic the Nail Psoriasis Severity Index (NAPSI) assessments were performed for the patient, which demonstrated a marked and progressive improvement in nail lesions (**Figure 3**).These favorable therapeutic outcomes were sustained stably during regular follow-up evaluations conducted over the subsequent months. Routine blood, liver and kidney function tests were performed every 4-week interval. At each follow-up visit, clinical inquiry regarding fever, cough, limb swelling or pain was conducted to screen for severe infection and thrombotic events. Notably, no treatment-related adverse events were reported throughout the entire course of therapy.

## Discussion

Diagnosis of nail diseases is often delayed, particularly for inflammatory nail conditions, which are extremely difficult to identify when patients present with no cutaneous manifestations and pathological changes are confined exclusively to the nails. Nail psoriasis is often difficult to differentiate from onychomycosis in clinical practice. Approximately 30% of patients with nail psoriasis are complicated by concurrent onychomycosis, and those with this comorbidity tend to present with significantly higher NAPSI scores (1, 7). Onychomycosis may not only promote the progression of nail psoriasis but also compromise therapeutic efficacy.

Systemic therapy is generally indicated for nail psoriasis involving more than three nails or severe nail dystrophy with a NAPSI score greater than 20 (8). Beyond objective disease severity, patient quality of life and functional impairment serve as critical determinants of treatment strategy, as functional impairment of the fingers substantially affects daily activities. In the present adolescent patient, extensive bilateral fingernail involvement severely compromised manual function, supporting the indication for systemic pharmacotherapy. Multiple traditional treatment modalities are available for nail psoriasis, yet each carries notable limitations for pediatric application. Intralesional injection delivers drugs directly to inflammatory sites and is generally safe when performed by specialized clinicians; however, this invasive procedure is associated with potential adverse effects including injection pain, transient numbness, hematoma, and nail fold atrophy or pigmentation, resulting in poor treatment adherence among pediatric patients and their caregivers (8, 9). Conventional systemic retinoids such as acitretin represent a classic therapeutic option but exert a slow onset of action with minimal early lesion improvement. Importantly, acitretin may further exacerbate nail fragility and structural dystrophy in patients with thin, brittle nail plates (8) Additionally, acitretin raises safety concerns in pediatric populations owing to its potential adverse impact on children’s skeletal growth and development, making it unsuitable for this case. Although methotrexate is considered safe for intermittent and short-term use in children according to AAD-NPF guidelines despite lacking formal FDA approval for pediatric psoriasis, its teratogenic potential precluded its administration in this adolescent patient (10, 11). Before treatment initiation, rigorous pretreatment safety evaluation was completed. Comprehensive baseline laboratory assessments, including hematological, hepatic, renal, and lipid profiling, together with standardized tuberculosis and hepatitis B screening, excluded active infection, organic dysfunction, and thromboembolic risk, establishing a solid safety foundation for targeted therapy.

Nail psoriasis is believed to stem from keratinocyte dysfunction triggered by signal transducer and activator of transcription 3 (STAT3) activation via the IL-23/Th17 axis. Selective inhibition of the JAK/STAT signaling pathway using JAK inhibitors can alleviate nail matrix lesions induced by key pro-inflammatory cytokines, including interleukin IL-2, IL-7, IL-15, and IL-23 (12). Among this class of agents, upadacitinib exerts its therapeutic effect by suppressing JAK phosphorylation and exhibits enhanced selectivity for JAK1, thereby exerting potent anti-inflammatory activity (13). As a selective JAK1 inhibitor approved by the FDA for multiple autoimmune and inflammatory disorders, upadacitinib offers rapid therapeutic efficacy and convenient oral administration (14–16).These advantages optimize long-term medication adherence in adolescent patients and render it the preferred treatment option in this case.

To date, only one case report has documented the successful treatment of nail psoriasis with upadacitinib in a female pediatric patient (15). Thus, our case provides additional evidence supporting the efficacy of upadacitinib in the management of nail psoriasis in the pediatric population. While clinical data specifically for nail psoriasis remain limited for upadacitinib, this agent has demonstrated a favorable benefit-risk profile in clinical trials for patients with alopecia areata (AA), psoriatic arthritis(PsA) and atopic dermatitis (AD) (17–19). It therefore represents a potentially effective therapeutic strategy for immune-mediated disorders including psoriasis.

Collectively, these findings underscore the clinical value of upadacitinib in ameliorating nail psoriasis-related morbidity and functional impairment in adolescents with refractory isolated nail psoriasis. As an oral, rapidly acting JAK1-selective inhibitor, upadacitinib confers distinct clinical advantages over conventional systemic agents and injectable biologics, including superior medication adherence and expedited amelioration of nail architectural abnormalities. Notably, the patient achieved near-complete lesion resolution after 20 weeks of treatment, with subtle residual nail changes remaining, consistent with the objective NAPSI score improvements observed throughout follow-up. Strict pretreatment risk stratification, including tuberculosis screening and comprehensive hematological, hepatic, renal, and coagulation profiling, as well as systematic on-treatment laboratory surveillance, was rigorously implemented to mitigate the known boxed warning risks of severe infection and thrombosis associated with upadacitinib.

Several limitations of this retrospective case warrant acknowledgment: the absence of post-treatment histopathological validation of inflammatory regression, lack of prospective rheumatological imaging and serological screening for subclinical psoriatic arthritis, and unavailable standardized quality-of-life evaluation further constrain the generalizability of the present findings. Long-term low-dose maintenance therapy and regular multidisciplinary follow-up have been established for the patient to sustain clinical remission and monitor for latent disease relapse or delayed adverse events. In summary, this clinical experience supports the favorable efficacy and tolerability of upadacitinib in treating pediatric refractory nail psoriasis. Further large-scale, prospective controlled studies are urgently required to establish age-specific standardized dosing regimens, validate long-term safety profiles, and consolidate the clinical applicability of upadacitinib in juvenile nail psoriasis management.

## Data Availability

The original contributions presented in the study are included in the article/supplementary material. Further inquiries can be directed to the corresponding author.
